# Minimally Invasive Resection of an Extradural Far Lateral Lumbar Schwannoma with Zygapophyseal Joint Sparing: Surgical Nuances and Literature Review

**DOI:** 10.1155/2014/739862

**Published:** 2014-09-18

**Authors:** Vítor M. Gonçalves, Bruno Santiago, Vítor C. Ferreira, Manuel Cunha e Sá

**Affiliations:** Neurosurgery Department, Garcia de Orta Hospital, Avenida Torrado da Silva, 2801-951 Almada, Portugal

## Abstract

*Introduction*. Spinal schwannomas are benign nerve sheath tumors. Completely extradural schwannomas of the lumbar spine are extremely rare lesions, accounting for only 0,7–4,2% of all spinal NSTs. Standard open approaches have been used to treat these tumors, requiring extensive muscle dissection, laminectomy, radical foraminotomy, and facetectomy. In this paper the authors present the case of a minimally invasive resection of a completely extradural schwannoma. Operative technique literature review is presented. *Material & Methods*. A 50-year-old woman presented with progressive complains of chronic right leg pain and paresthesia. The magnetic resonance imaging revealed a giant well-encapsulated dumbbell-shaped extradural lesion at the L3-L4 level. The patient underwent a minimally invasive gross total resection of the tumor using a tubular expandable retractor system. *Results*. The patient had complete resolution of radiculopathy in the immediate postoperative period and she was discharged home, neurologically intact, on the second postoperative day. Postoperative MRI demonstrated no evidence of residual tumor. At latest follow-up (18 months) the patient remains asymptomatic. *Conclusion*. Although challenging, this minimally invasive procedure is safe and effective, being an appropriate alternative, with many potential advantages, to the open approach.

## 1. Introduction

Schwannomas are nerve sheath tumors (NSTs) that originate from neural crest-derived Schwann cells mainly located along the dorsal sensory spinal roots and only sporadically arising from the ventral motor roots. They represent around 85% of all NSTs, which are the most common form of spinal cord tumor, corresponding approximately to 30% of all primary spinal neoplasms and having roughly the same incidence as meningiomas [1].

Schwannomas are considered slow growing, benign lesions, although malignant subtypes exist. These tumors are uniformly distributed throughout the spine and topographically, they may be intradural-extramedullary (72%), intra- and extradural (13%), completely extradural (13%), or, exceptionally, intramedullary (1%) [2–4]. Purely intraosseous vertebral localization has also been described in the literature, constituting the rarest pattern and accounting for less than 0,2% of primary bone tumors [2, 5].

Being unusual, completely extradural spinal schwannomas that are located in the lumbar spine form an even rarer subgroup of spinal NSTs, representing only 0,7 to 4,2% of all extradural schwannomas [1, 4]. This may be justified by the longer distance lumbar nerve roots having to travel before reaching the intervertebral foramen, when compared to cervical or thoracic roots [6].

In current literature, completely extradural schwannomas have not been methodically addressed nor given much attention due to their rarity, making it difficult to establish optimal surgical strategies for their treatment [2, 7]. Accounting for only 2,4 to 3,2% of all spinal NSTs, they have always been included in the same standard treatment approaches used for all schwannomas, in spite of being considered a special issue due to their unique morphology and compartmental peculiarities [2, 4].

A minority subgroup of these tumors exhibits an invasive pattern [8]. According to their dimension, extension, and invasiveness, they may have contiguous intraspinal, foraminal, extraforaminal, and intraosseous components [9, 10]. A bony constriction at the foramen gives them an hourglass shape, being described as dumbbell tumors [3].

In the literature, the term giant spinal schwannoma is not well defined [8]. To simplify and solve this problem, Sridhar et al. advocated a classification system where giant spinal schwannomas are defined as those that extend over more than two vertebral levels (type II), those that have an extraspinal extension of more than 25mm (giant dumbbell type IVb), and those lesions that erode the vertebral bodies and extend posteriorly and laterally into the myofascial planes (giant invasive tumors, type V) [8]. A “modified Sridhar classification of benign NSTs” has also been proposed including intraosseous schwannomas [10].

Presentation is usually between the third and fifth decades of life, with no particular gender predilection [11]. The gold standard treatment is surgery and the goal is gross total resection (GTR), with preservation of the involved nerve root, which is generally curative and has minimal associated morbidity [12]. Recurrences are rare after GTR, except in cases of neurofibromatosis. The long-term recurrence rate is 6% to 12% with a mean time recurrence of 5 years.

In this paper we present a minimally invasive resection of an extradural lumbar schwannoma with contiguous intraspinal, foraminal, and extraforaminal components. Operative technique and surgical nuances are described. A revision of current literature is discussed.

## 2. Material & Methods

### 2.1. Case Report

#### 2.1.1. History

A 50-year-old woman, without known history of neurofibromatosis, was admitted with progressive complains of right distal anterior thigh and medial lower leg pain and paresthesias for 2 years and recently proximal ipsilateral leg weakness. These symptoms were refractory to medical therapy, exacerbated in the night and by movements, causing severe functional disability with progressing shortening of walking performance. Neurological examination revealed an abolished right patellar reflex and a 4+/5 strength in the ipsilateral psoas muscle. There were no other sensory-motor deficits or sphincter (bowel/bladder) dysfunction.

#### 2.1.2. Imaging

**Figure 1:**
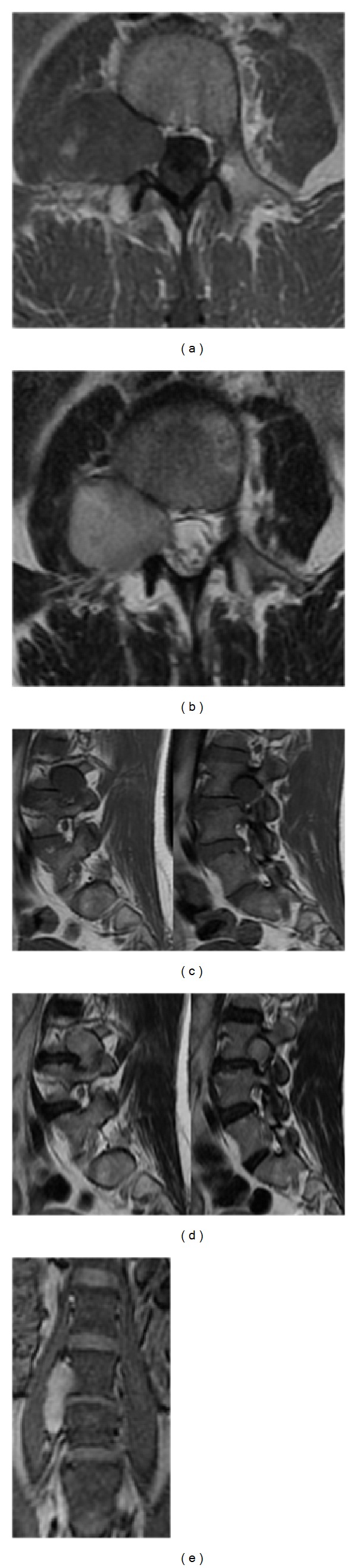
Preoperative MRI documents a right L3/L4 completely extradural dumbbell-shaped tumor with extraforaminal extension into the right psoas muscle: (a) T1-weighted; (b) axial T2-weighted with gadolinium enhancement; (c) sagittal T1-weighted showing extraforaminal and foraminal tumor components; (d) sagittal T2-weighted with gadolinium enhancement showing extraforaminal and foraminal tumor components; (e) coronal view with gadolinium.

On MRI, an expansive, well-circumscribed, encapsulated dumbbell-shaped extradural lesion, 36 × 26 × 33 mm (axial × sagittal × coronal), was seen, at the right L3-L4 level (Figure 1). This globoid mass was T2W hyperintense, T1W hypointense, and heterogeneously enhanced after gadolinium injection. It presented with contiguous intraspinal, foraminal, and extraforaminal components and remodeling of the neural foramen and psoas muscle due to its extension to the paravertebral region, giving the tumor an hourglass shape. On the MRI there was an erosive but not infiltrative or invasive lesion. No dural enhancement was seen.

#### 2.1.3. Operative Technique

**Figure 2:**
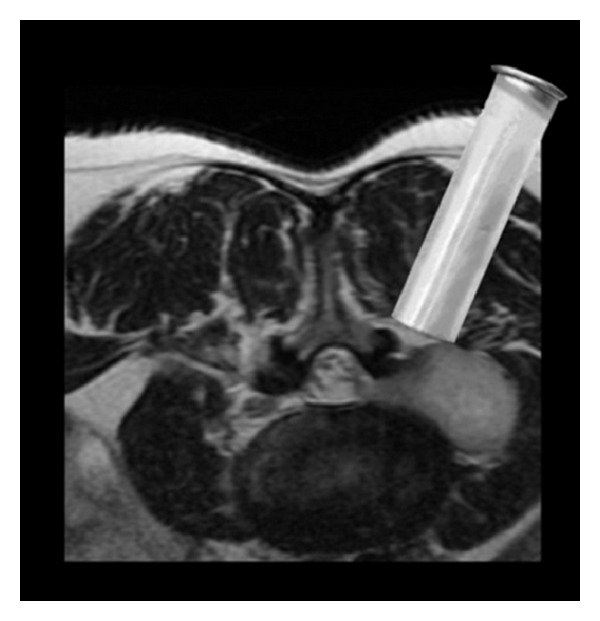
Resection of a completely extradural lumbar schwannoma through a minimally invasive approach using an expandable transmuscular tubular retractor, positioned laterally to the facet joint complex.

The patient was electively operated by one of the senior authors (Bruno Santiago) using a minimally invasive approach with an expandable tubular retractor system (Figure 2).

**Figure 3:**
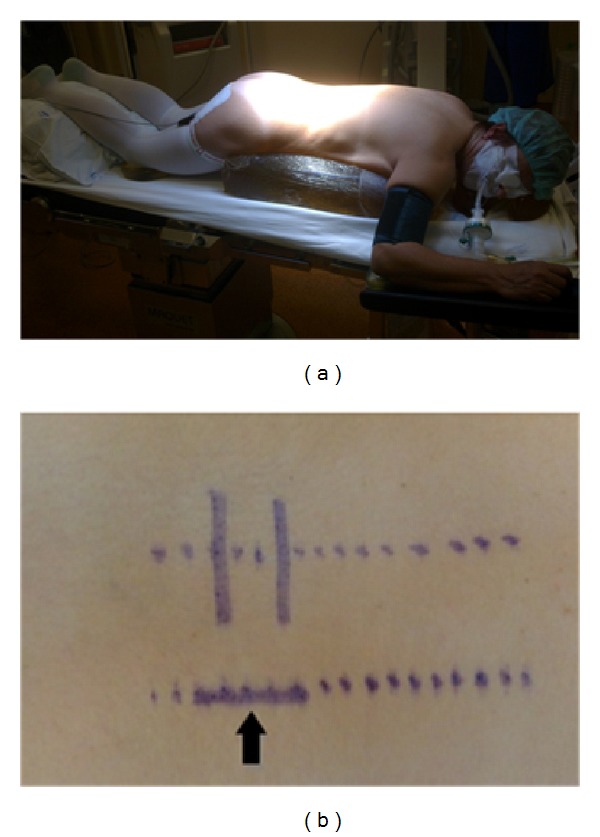
(a) Patient positioning and (b) preoperative planned skin incision (arrow).

*Anesthesia and Positioning*. Under general anesthesia the patient was intubated. Arterial line was placed for blood pressure monitoring. Intravenous dexamethasone and antibiotic prophylaxis (cefazolin 1 g) were administered preoperatively. The patient was positioned prone (Figure 3(a)). All areas under pressure were padded. The posterior lumbosacral area was subsequently sterilized and draped after the skin was dried.

**Figure 4:**
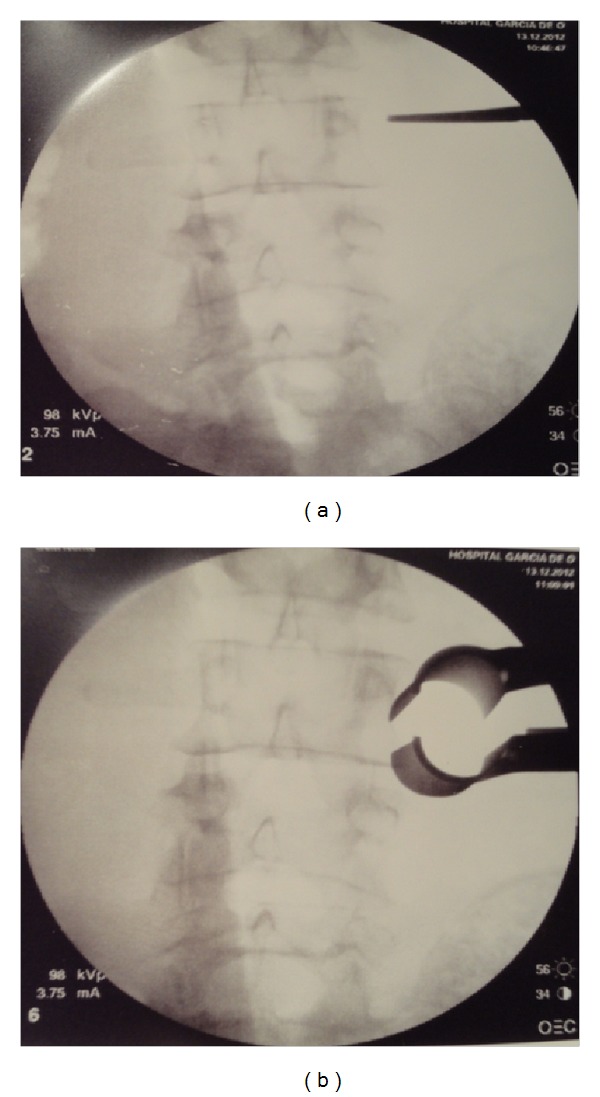
(a) Intraoperative fluoroscopic localization of L3/L4 extraforaminal space; (b) retractor placement and angulation into the intertransverse space, lateral to the L3/L4 facet complex.

**Figure 5:**
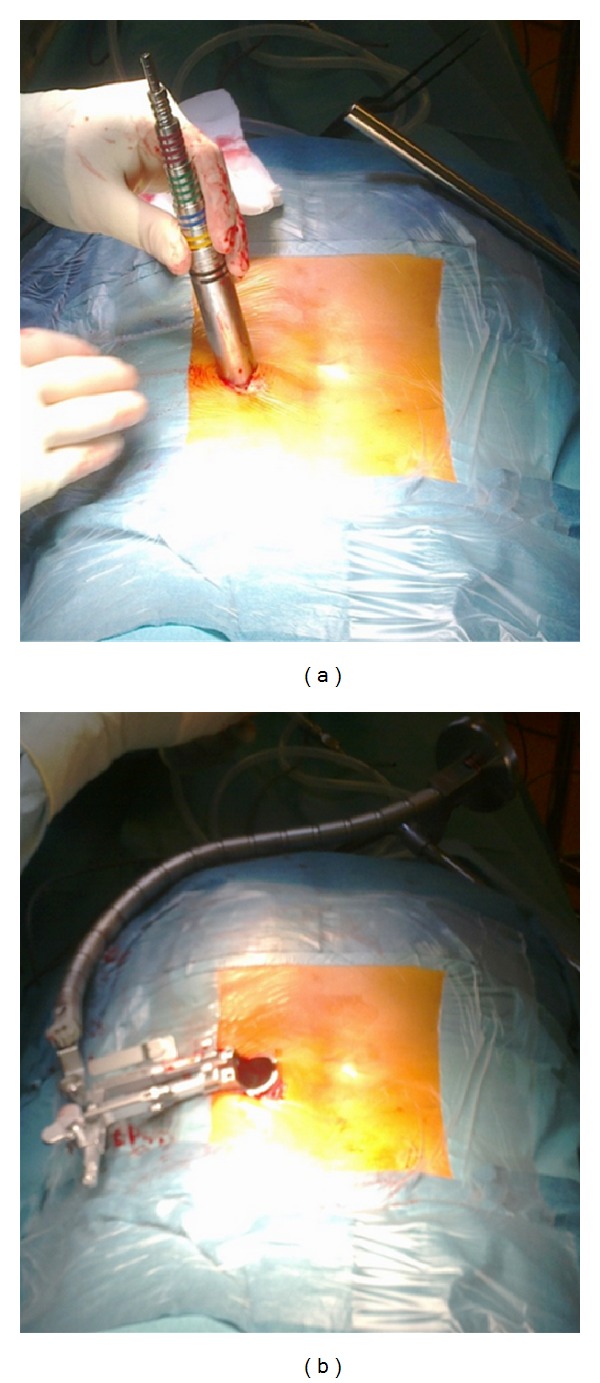
(a) Sequential muscle dilation and (b) expandable tubular retractor fixation to the articulated arm.

*Posterior Lumbar Minimally Invasive Approach*. The C-arm fluoroscope was brought into the field for localization of the L3-L4 extraforaminal space (Figure 4(a)). A 3 cm right paramedian longitudinal skin incision, 4 cm lateral to the midline, facilitated surgical instruments to be correctly angulated to directly access the ipsilateral L3-L4 extraforaminal space (Figure 3(b)). The lumbosacral fascia was identified and incised along the predictable dilator track. The initial dilator was inserted through the incision and fluoroscopically directed toward the right L3-L4 facet complex and docked on the L3 isthmus. The tip of this initial dilator was utilized to palpate the bony anatomy helping to identify proper dilator positioning. Once this was accomplished, dilators were sequentially introduced for paraspinal muscle dilation (Figure 5(a)). An expandable tubular retractor was inserted over the dilators and firmly settled in place with the flexible and articulated arm (Figure 5(b)). The retractor blades were opened in a rostral-caudal fashion and properly angulated into the intertransverse L3-L4 space, lateral to the right L3 isthmus and L3-L4 facet joint. Fluoroscopy system was used to verify the correct positioning of the retractor (Figure 4(b)). At this stage, the operating microscope was brought into the field for visualization.

**Figure 6:**
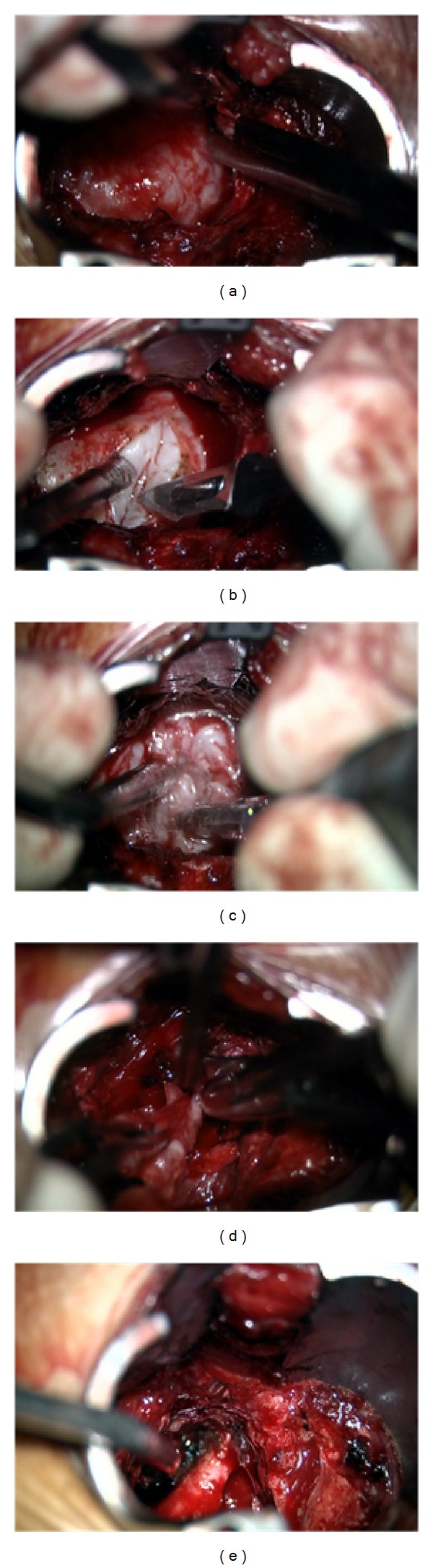
Intraoperative microsurgical images showing (a) bone removal allowing tumor exposition; (b) tumor capsule incision; (c) internal debulking with CUSA; (d) piecemeal tumor removal; (e) L3 nerve root preservation after tumor gross total resection.

*Microsurgery Technique*. Under microscope view, residual soft tissue was removed from over the dorsal cortical surface of the adjacent lumbar lateral masses. The intertransverse membrane was opened allowing access to the tumor capsule. The tumor capsule was then exposed and carefully inspected to ensure that there was no nerve root passing by. Tumor capsule coagulation was then performed, followed by internal debulking to allow mobilization of the capsule, facilitating extracapsular dissection, between the tumor and the psoas muscle. Internal debulking and piecemeal removal of the tumour was achieved with a bipolar coagulation and a Cavitron ultrasonic surgical aspirator (CUSA) (Figures 6(b) and 6(c)). In the specific case of extradural schwannomas, the nerve fascicles are distinct from the tumor, allowing a plane of dissection between tumor and nerve fibers to be established (Figure 6(d)). The tumor was meticulously and gently dissected from the attached nerve root, revealing a plane between them, until the nerve root lied free within the visual field (Figure 6(e)). Intraoperative neural stimulation is useful to ensure that no viable nervous structures are harmed throughout the surgery, although it was not used in this case. Gross total resection was accomplished, and adequate L3 nerve root decompression was documented. Closure was performed in layered fashion.

## 3. Results

GTR of this giant completely extradural lumbar schwannoma was performed without sacrificing any nerve root. The completely extradural location of the tumor, as previewed on the preoperative diagnostic images, was corroborated with the intraoperative findings. There were no procedure-related complications. The operative time was 170 minutes and the estimated total blood loss was 300 mL without transfusion requirements.

**Figure 7:**
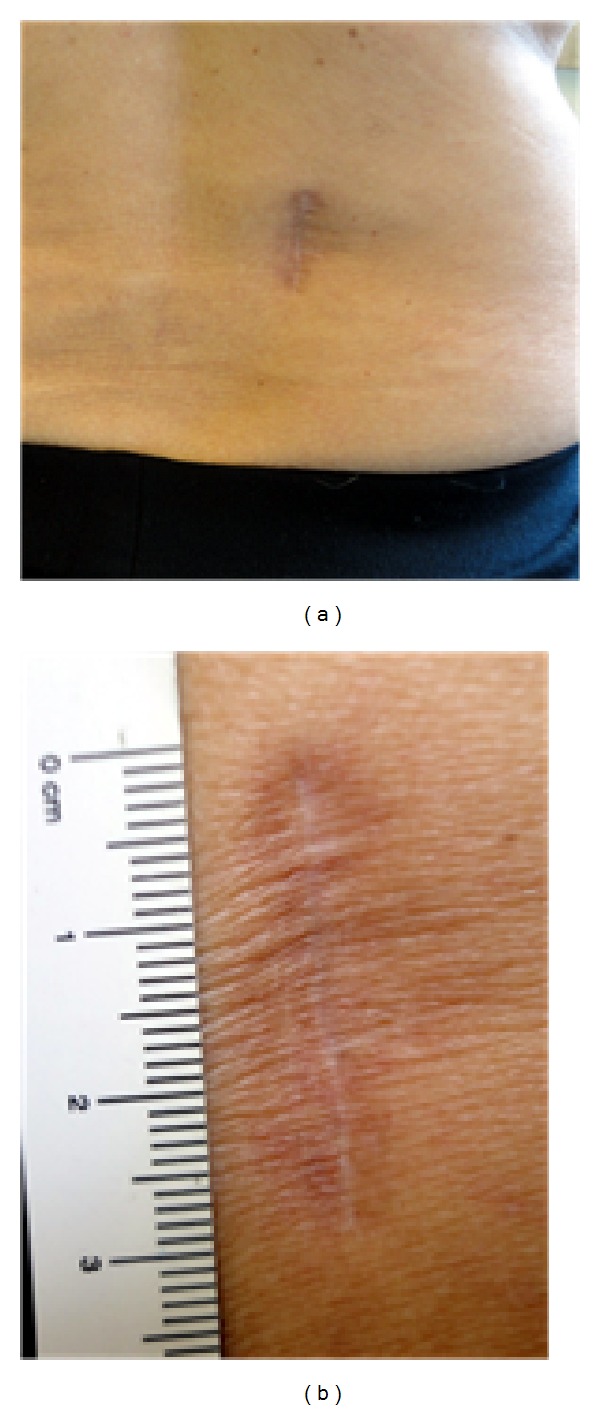
(a) Three cm long right paramedian longitudinal skin incision 4 to 5 cm off the midline; (b) final cosmetic result.

In the immediate postoperative period the patient had complete resolution of radiculopathy and presenting neurological deficit. She was discharged home, neurologically intact, on the second postoperative day. She was observed postoperatively for a clinical assessment at one week and, subsequently, 1, 3, 6, and 12 months after surgery (Figure 7). A follow-up MRI was performed 6 months after the surgical procedure. The patient returned to regular activities within 4 weeks.

**Figure 8:**
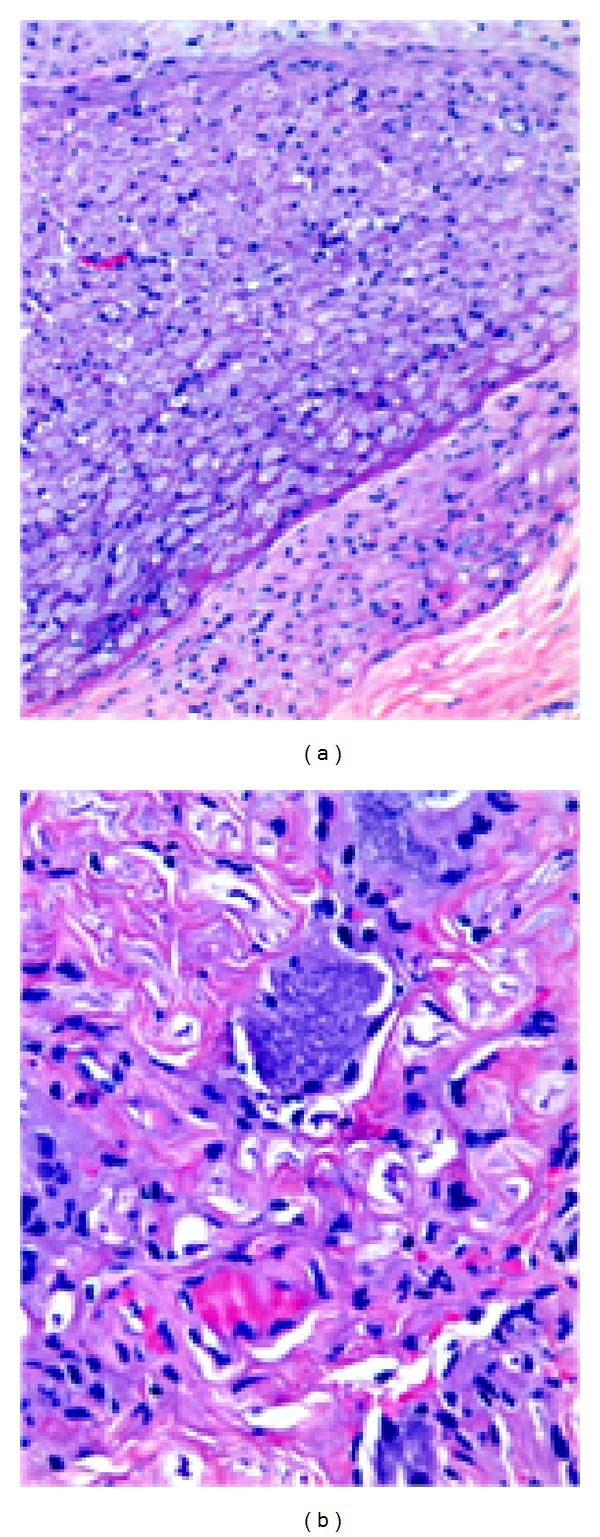
(a and b) Biphasic pattern with cellular Antoni A and hypocellular Antoni B areas. Compact fascicles of elongated tumor cells with slight nuclear polymorphism. Hyalinized vessels.

Histopathological analysis of the resected lesion demonstrated a benign extradural dumbbell schwannoma (WHO grade I) (Figure 8). The pathological tissue was found to be external to the dural sac and totally outside the nerve root, which was not definitely abnormal in appearance (Figure 6(e)).

**Figure 9:**
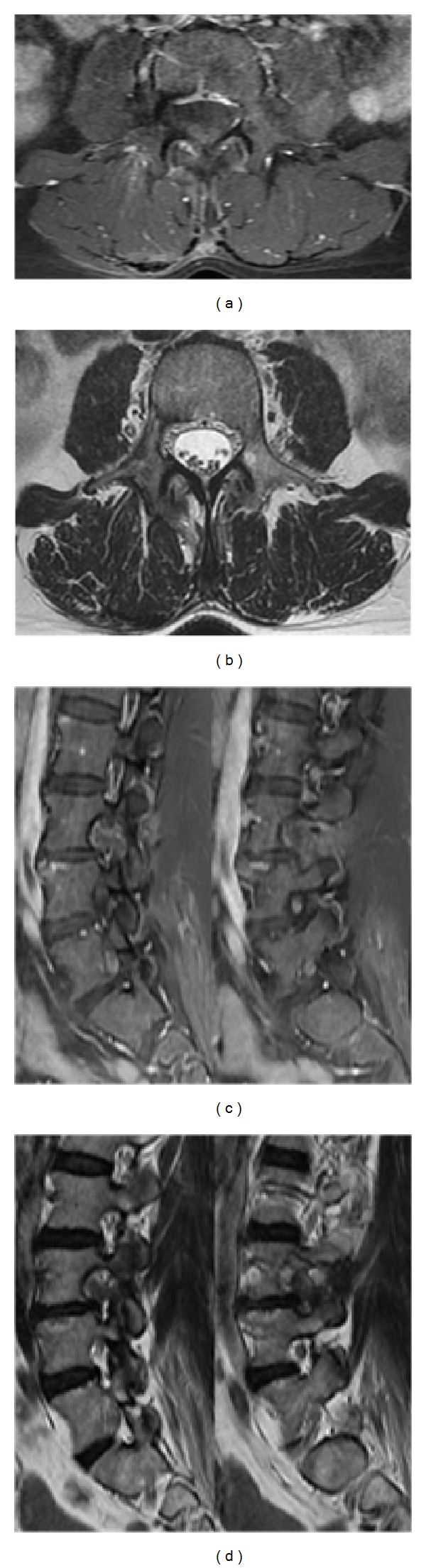
Postoperative MRI demonstrates gross total resection of the tumor and postoperative changes with no muscle atrophy: (a) axial T1-weighted with gadolinium enhancement; (b) T2-weighted; (c) sagittal T1-weighted with gadolinium enhancement; (d) T2-weighted.

The postoperative MRI (6 months after surgery) showed no evidence of residual tumor (Figure 9). At 18-month follow-up the patient remains asymptomatic.

**Figure 10:**
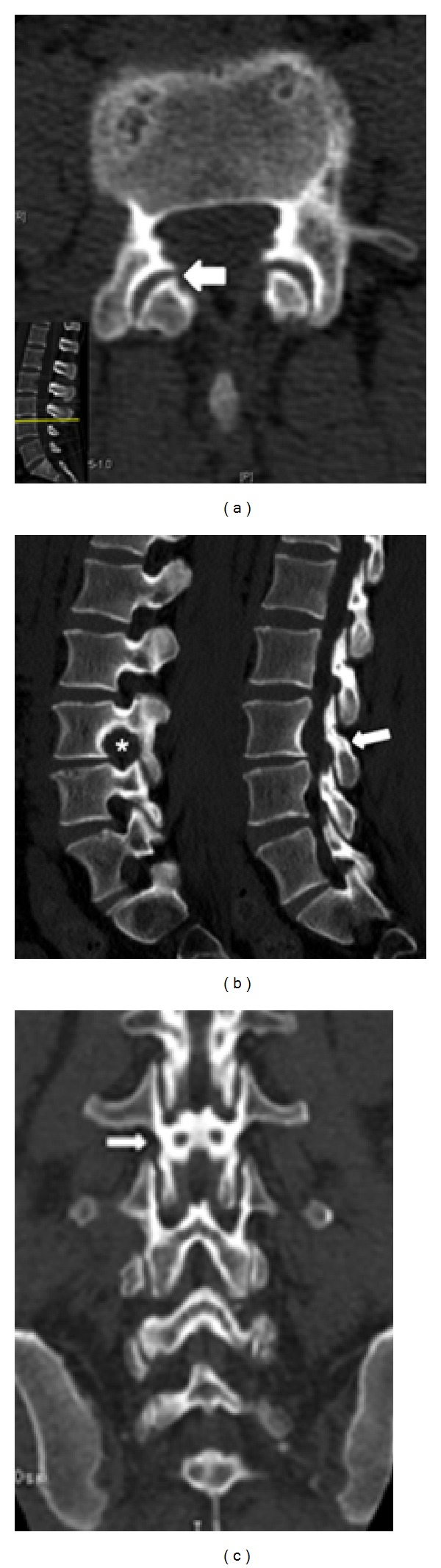
Postoperative bone window CT scan: (a) axial view demonstrating facet joint integrity (arrow); (b) sagittal view showing widening of the neural foramen (∗) and pars interarticularis sparing (arrow); (c) coronal view also illustrating pars preservation (arrow).

Postoperative bone window computed tomography (CT) scan confirmed pars interarticularis and facet joint integrity (Figure 10). At 18-month follow-up the patient remains asymptomatic.

## 4. Discussion

Completely extradural spinal schwannomas constitute a rare subgroup of spinal NSTs. In current literature, they have not been methodically addressed, making it difficult to establish optimal surgical strategies for their treatment [4]. Most of the papers sporadically published about extradural NSTs do not explicitly evoke the nature of the nerve root involvement: whether it is “adherent to” or “enfolded in” the pathological tissue. In this particular clinical case, the pathological tissue was not only completely external to the unviolated dural sac, but also outside the nerve root which was carefully preserved. The tumor was only “attached” or “adherent” to the root, with a clear dissection plane between the tumor and nerve fibers. This is what defines a completely extradural schwannoma.

As described by Celli et al. in their retrospective series of 24 extradural schwannomas surgically treated, these tumors rarely localize in the lumbosacral region and generally extend for more than one vertebral segment and occupy a lateral position in the spinal canal, nearly always with an intraspinal-extraspinal hourglass shape [4].

Although different classifications for dumbbell tumors of the spine have been proposed, none is sufficient to determine optimal surgical planning in view of recent advances in minimally invasive spine surgery techniques [9]. The treatment of extradural schwannomas is challenging. Biomechanical reasoning should always be kept in mind and guide the surgical route, in order to avoid iatrogenic complications. Nevertheless it is important to stress that fear of destabilizing the spine should not compromise the exposure required to safely remove these tumors. Supplemental stabilization may be required but must be reserved for the treatment of giant invasive schwannomas (Sridhar type V), extending into the vertebral bodies and paravertebral region, or for those that extend over more than two vertebral levels (Sridhar type II), in which a more radical bone removal would be necessary to completely resect the tumor. This is particularly applicable for surgery performed across the cervicothoracic or thoracolumbar junctions. For other types of dumbbell schwannomas, like the one we present, with an extraspinal extension of more than 25 mm (Sridhar type IVb), minimally invasive techniques are recommended.

The optimal surgical approach may depend on many factors. Tumor related factors include location (dorsal, ventral, or lateral), extension (intraspinal, foraminal, and/or extraforaminal components, contiguous vertebral segments involved), affected neural compartments (intradural-extramedullary, intraextradural, purely extradural, intramedullary, or intraosseous), size, and “modified Sridhar classification of benign NSTs” (nongiant, giant). Patient related factors include the preoperative neurological status (presence or not of neurologic deficit), age of the patient, and the duration of symptoms. Surgeon's related factors combine an understanding of the underlying anatomy, surgeon's experience, and individual preference.

The mainstay of treatment for extradural schwannomas is, like in other NSTs, GTR which is usually curative. According to published surgical series, conventional open posterior midline approaches have typically been used to treat extradural schwannomas, requiring a dorsal midline incision, wide-ranging bilateral disruption to the paraspinal muscles and ligaments (major contributors to the maintenance of proper spine biomechanics), extensive laminectomy (from one or two levels above and below the tumor), radical foraminotomy, and complete unilateral facetectomy [7, 10, 13, 14]. Fusion may be necessary for stabilization, in order to prevent pain, spinal deformity, instability, and neurological deterioration [6, 15–22].

To avoid these iatrogenic complications, many reports describe the resection of spinal tumors (intradural-extramedullary and intradural-intramedullary) through a more restricted hemilaminectomy and medial facetectomy (resection up to one-third of the medial facet joint) [23, 24]. With the advent of newer technology (advances in microscopy, tubular retractors, and other specialized instruments), combined with the upgrade of imaging systems (both in and out of the operating room), and an improved understanding of surgical anatomy and biomechanics of the spine, advanced minimally invasive approaches have been developed and refined in the last decade. In recent reports, minimally invasive techniques have been successfully used on the resection of intradural spinal tumors, and the results, when compared with standard open approaches, demonstrated reduced soft tissue destruction (muscle atrophy and denervation), blood loss, and length of hospitalization [13, 18, 24–27].

Few reports exist stressing the potential advantages of minimally invasive techniques in the treatment of extradural schwannomas (Table 1). Good surgical results have been recently reported demonstrating feasibility, safety, and effectiveness of these approaches in the resection of giant extradural schwannomas, using expandable and nonexpandable tubular retractors [3, 6, 14, 28].

Lu et al. presented a retrospective analysis of three patients with extradural lumbar schwannomas of the lumbar spine who successfully underwent miniopen resection and fusion using expandable tubular retractors. Two of the three tumors were giant according to Sridhar classification. Two patients had undergone prior operations (circumferential fixation and discectomy). In these cases the main advantage was to approach the tumor lateral to the scar tissue, probably reducing time required for surgical dissection and risk of complications. GTR was achieved in all patients but one in whom a subtotal resection (STR) was intentionally done to preserve a functional nerve root avoiding* de novo* neurological deficit and/or deafferentation pain. Fusion was achieved in all patients with no perioperative complications. All the patients had short hospitalization (even after reoperation) and operative time [14].

Haji and colleagues recently published their retrospective case series for the treatment of extradural, intradural-extramedullary, and intramedullary spinal tumors using minimally invasive techniques (with expandable tubular retractors). Six patients had completely extradural schwannomas (5 in the lumbar spine and 1 thoracic) that were resected with good results. The size of the tumors was not referred to. GTR was achieved in all patients but one (a small amount of residual tumor was documented in postoperative images), with comparable results in terms of blood loss, operative time, hospitalization days, and narcotic usage, when compared to prior reported data with standard open techniques [25, 28, 29]. With increasing experience, reduced operative time, blood loss, complications, length of hospital stay, postoperative pain, and spinal instability might be expected [25].

Weil et al. reported the case of a minimally invasive removal of a giant extradural foraminal schwannoma through a nonexpandable tubular retractor. According to authors' point of view, this may be associated with even less tissue destruction than miniopen techniques using expandable retractors, translating into less blood loss and a quicker functional recovery. GTR was achieved and no complications were found [6].

Nzokou and colleagues recently presented a retrospective series of consecutive patients who underwent minimally invasive resection of spinal tumors using nonexpandable tubular retractors. Seven extradural schwannomas were removed, 5 in the thoracic and 2 in the lumbar spine. In all patients a GTR was achieved except in one thoracic tumor, in which the tumor capsule was adherent to the diaphragm. A STR (90% of tumor removal) was performed in this patient. Authors did not mention the dimensions of the tumors [28].

To our knowledge, the case we present is the 19th completely extradural schwannoma resected through a minimally invasive approach, the 13th in the lumbar region and, considering its size and Sridhar modified classification, the 4th giant tumor.

## 5. Conclusion

For this kind of pathology, consensus regarding the best treatment approach does not exist. This minimally invasive approach which technically is not extrademanding allows achieving results equivalent or superior to traditional open technique. Completely resecting extradural schwannomas can be both technically challenging and rewarding because these tumors are most often benign lesions for which curative resections can be safely performed through minimally invasive approaches. Patient outcomes are usually very good. Minimally invasive approaches provide adequate exposure to safely remove giant dumbbell type extradural schwannomas, without the need for potentially destabilizing facet or pedicle resection. Tubular retractors provide a direct surgical corridor to the tumor minimizing soft tissue damage and allowing decreased neural structures retraction or manipulation. To assure optimal postoperative outcome and patient satisfaction, proper patient selection is paramount.

This minimally invasive procedure is safe and successful, being an appropriate alternative, with many potential advantages, to the open approach for the treatment of giant lumbar extradural schwannomas. Further studies are needed to completely validate this.

## Figures and Tables

**Table 1:** Literature review of patients with completely extradural spinal schwannomas operated through minimally invasive approaches.

Author YOP	Case	Age sex	Level	Tumor size	Preop. symptoms	Preop. deficit	Resection	Fusion	Blood loss (mL)	OR time (min)	LOS (days)	Postop. symptoms	Outcome	Complications
Lu et al., 2009 [14]	1	49/M	L2	Giant	LBP, LEP	—	GTR	L1-L2	400	270	4	Resolved	Resolved	None
“	2	48/M	L3	Nongiant	LBP, LEP	—	GTR	L3-L5	250	150	3	Improved	Improved	None
“	3	57/M	L5	Giant	LBP, LEP	Foot drop	STR	L5-S1	100	180	3	Resolved	Improved	None
Haji et al., 2011 [25]	4	61/ F	L5	?	LEP	L5 sensory deficit	GTR	No	500	260	1	Resolved	Resolved	None
“	5	27/M	L1	?	LBP, LEP	—	STR	No	500	264	3	Resolved	Resolved	None
“	6	30/F	L4	?	LEP, LEN, GI	LEH, atrophy	GTR	No	500	270	2	Persistent LEN	Improved	None
“	7	56/M	L5	?	LEP	—	GTR	No	1200	285	2	Resolved	Resolved	None
“	8	47/F	T4	?	LEP, LEN, LEW, GI	LEW, H, spasticity	GTR	No	1250	225	4	Resolved	Resolved	None
“	9	64/F	L3-4	?	LEP, SD	LEA	GTR	No	600	250	1	Improved	Improved	None
“	10	26/M	L1-L2	?	LBP	—	GTR	No	1100	210	1	Resolved	Resolved	None
Weil et al., 2011 [6]	11	77/F	L3	Giant	LBP, LEP	LEW	GTR	No	200	180	2	Resolved	Resolved	None
Nzokou et al., 2013 [28]	12	64/M	T11-12	?	—	T12 H	STR	No	500	540	4	Resolved	Improved	None
“	13	45/F	T6-7	?	—	—	GTR	No	200	160	3	Resolved	Improved	None
“	14	68/F	T3-4	?	—	—	GTR	No	100	85	1	Resolved	Improved	None
“	15	76/F	L3-S1	?	—	—	GTR	No	200	180	2	Resolved	Improved	None
“	16	34/M	T10-11	?	—	—	GTR	No	50	105	1	Resolved	Improved	None
“	17	69/M	T8-9	?	—	—	GTR	No	50	75	1	Resolved	Improved	None
“	18	72/F	L3-4	?	LBP, LEP	—	GTR	No	100	150	2	Resolved	Improved	None
Present case 2014	19	50/F	L3	Giant	LBP, LEP, LEN, LEW	LEW, Patellar A	GTR	No	300	170	2	Resolved	Improved	None

YOP: year of publication; OR: operating room; LOS: length of stay; LBP: low back pain; LE: low extremity; P: pain; N: numbness; W: weakness; H: hypoesthesia; A: areflexia; SD: sphincter disturbance; GI: gait impairment; GTR: gross total resection; STR: subtotal resection.
